# Exploring the diversity of virulence genes in the *Magnaporthe* population infecting millets and rice in India

**DOI:** 10.3389/fpls.2023.1131315

**Published:** 2023-05-09

**Authors:** K. B. Palanna, H. D. Vinaykumar, S Koti. Prasanna, H. Rajashekara, B. N. Devanna, C. Anilkumar, B. Jeevan, H. R. Raveendra, Farooq Khan, C. H. Sai Bhavana, Vinod Upadhyay, T. S. S. K. Patro, Laxmi Rawat, M. Rajesh, P. T. Saravanan, Prahlad Netam, G. Rajesha, I. K. Das, H. E. Patil, A. K. Jain, S. Saralamma, S. Chandra Nayaka, G. Prakash, T. E. Nagaraja

**Affiliations:** ^1^ ICAR-All India Coordinated Research Project (ICAR-AICRP) on Small Millets, PC Unit, University of Agricultural Sciences, Gandhi Krishi Vigyana Kendra (GKVK), Bengaluru, Karnataka, India; ^2^ Department of Plant Biotechnology, University of Agricultural Sciences, Gandhi Krishi Vigyana Kendra (GKVK), Bengaluru, Karnataka, India; ^3^ Department of Plant Pathology, Vivekananda Parvatiya Krishi Anusandhan Sansthan, Almora, Uttarakhand, India; ^4^ ICAR-National Rice Research Institute, Cuttack, Odisha, India; ^5^ ICAR-All India Coordinated Research Project (ICAR-AICRP) on Small Millets Zonal Agril. Research Station, Vishweshwaraiah Canal (V.C.) Farm, Mandya, Karnataka, India; ^6^ Regional Agricultural Research Station, Assam Agriculture University, Gossaigaon, Assam, India; ^7^ Department of Plant Pathology, Agricultural Research Station, Gajularega, Vizianagaram, Andra Pradesh, India; ^8^ Department of Plant Pathology, Uttarakhand University of Hort. and Forestry, Ranichauri, Uttarakhand, India; ^9^ Department of Plant Pathology, Center for Excellence in Millets, Athiyandal, Tiruvannamalai, Tamil Nadu, India; ^10^ Department of Plant Pathology, Zonal Agricultural Research Station, Kumharwand Farm, Jagdalpur, Chhattisgarh, India; ^11^ Indian Council of Agricultural Research ICAR-Indian Institute of Millets Research, Rajendranagar, Hyderabad, Telangana, India; ^12^ Hill Millet Research Station, Navasari Agricultural University, Waghai, Dangs, Gujarat, India; ^13^ Department of Plant Pathology, College of Agriculture, Rewa, Madhya Pradesh, India; ^14^ ICAR-All India Coordinated Research Project (ICAR-AICRP) on Small Millets, Regional Agricultural Research Station, Nandyal, Andhra Pradesh, India; ^15^ Institute of Excellence, Vijnana Bhavan, University of Mysuru, Manasagangotri, Karnataka, India; ^16^ ICAR-Indian Agricultural Research Institute, New Delhi, India

**Keywords:** millet crops, Magnaporthe, virulent and avirulent genes, blast pathogen, PCR

## Abstract

Blast pathogen, *Magnaporthe* spp., that infects ancient millet crops such pearl millet, finger millet, foxtail millet, barnyard millet, and rice was isolated from different locations of blast hotspots in India using single spore isolation technique and 136 pure isolates were established. Numerous growth characteristics were captured *via* morphogenesis analysis. Among the 10 investigated virulent genes, we could amplify MPS1 (TTK Protein Kinase) and Mlc (Myosin Regulatory Light Chain edc4) in majority of tested isolates, regardless of the crop and region where they were collected, indicating that these may be crucial for their virulence. Additionally, among the four avirulence (*Avr*) genes studied, *Avr-Pizt* had the highest frequency of occurrence, followed by *Avr-Pia*. It is noteworthy to mention that *Avr-Pik* was present in the least number of isolates (9) and was completely absent from the blast isolates from finger millet, foxtail millet, and barnyard millet. A comparison at the molecular level between virulent and avirulent isolates indicated observably large variation both across (44%) and within (56%) them. The 136 *Magnaporthe* spp isolates were divided into four groups using molecular markers. Regardless of their geographic distribution, host plants, or tissues affected, the data indicate that the prevalence of numerous pathotypes and virulence factors at the field level, which may lead to a high degree of pathogenic variation. This research could be used for the strategic deployment of resistant genes to develop blast disease-resistant cultivars in rice, pearl millet, finger millet, foxtail millet, and barnyard millet.

## Introduction

Millets are one of the earliest food crops that humans have grown, but due to urbanization and industrialization, they were neglected in favor of wheat and rice, which are most widely consumed as food and forage crops in the semi-arid regions and is highly crucial for the global food security and farmers livelihood. Globally, 30.73 million tons of these ancient millets are produced, and of which 11.42 million tons (or 37% of global production) is contributed by India (http://www.fao.org). These millets are considered as the most promising grains for preventing hunger and malnutrition besides the guaranteeing their widespread access across the globe (Gupta, 2006). Considering the importance of millets to the farmers’, consumers and the environment, Government of India has laid an initiative for popularization of these crops by celebrating the international year of Millets, through the United Nations, and this has garnered support from many countries. The support from Government of India in promoting millets production and increasing the area under millets reflects that these are the future crops for nutritional and food security.

Production and productivity of millets has been majorly succumbing to various abiotic and biotic factors that are set to be increasing day by day due to present global climate change scenario. The blast disease, which is incited by *Magnoporthe* spp., is a most destructive biotic constraint affecting the millets, including rice. It affects in all the growth stages of plants especially different aerial parts of the plants, from the seedling stage to seed setting in finger millet, causing neck and/or finger blast, while it is restricted to the leaf only in the case of pearl millet, foxtail millet, and barnyard millet (Palanna et al., 2021; Rajashekara et al., 2021). Blast is found in most of the regions where millets are grown. According to Nagaraja et al. (2007), yield loss in finger millet due to this disease ranges from 28 to 36%, and can reach as high as 80 to 90% in some locations. Panicle blast is the most severe form of this disease, and damages the crop at all growth phases, from seedling to grain formation (Takan et al., 2004 and Babu et al., 2013). More than 50 graminaceous hosts, including rice, wheat, finger millet, pearl millet, and foxtail millet are susceptible to *M. grisea* (Ou, 1985 and Rossman et al., 1990). Considerable yield loss of pearl millet grain (Timper et al., 2002) and forage (Wilson and Gates, 1993) has been reported due to blast disease and it has occurred as a serious constraint-causing huge yield loss in both grain and fodder of pearl millet hybrids in India (Lukose et al., 2007; Anonymous, 2009). The yield loss as high as 70 to 80 per cent has been reported in blast disease, when the predisposition factors like temperature (25-27 °C), high humidity (>85%), excessive use of nitrogen fertilizer favored the blast diseases epidemics crop (Piotti et al., 2005). Further, the disease is also on rise in majority of the foxtail millet growing regions of India.

The disease severity depends both on the favorable environment factors and also on the pathogen virulence. However, there is lack of information on the diversity of virulence and avirulent gene in *Magnoporthe* spp., pathogen populations infecting millets in India. However, efforts are put forth to know the aggressiveness and the genetic diversity of the blast pathogen populations (Takan et al., 2012; Babu et al., 2013). Lack of knowledge on finger millets and other millets -blast pathosystem, genetic diversity of the infected millet population, and host plant resistance (HPR) has hampered the efforts to create and deploy blast-resistant cultivars that are adaptable to various agro-ecological systems. However, the morpho-molecular characterization, and genetic diversity analysis of *avirulence* genes in the *Magnaporthe oryzae* in rice was studied using different molecular markers (Yadav et al., 2019; Amoghavarsha et al., 2022) Hence, an effort has been made to use specific genetic markers linked to specific trait to know the genetic diversity of virulent and avirulent genes in the *Magnaporthe* population that infects the millets and rice crops throughout India.

In this study, diversity and distribution of 10 virulent (*vir*) genes (*ERG2, Cut1, PWL2, MPG1, MPS1, Slp1, Exo70, ABC1, Mlc* and *Tps1*) and four avirulent (*Avr*) genes (Z41/Z42, Z23/Z24, YL169/YL149, Z21/Z22 and Z27/28) has been analyzed in 136 blast isolates infecting millets, such as finger millet, barnyard millet, foxtail millet and pearl millet, and rice across the different millet occupied regions in India.

## Material and methods

### Study area and sampling collection

**Figure 1:**
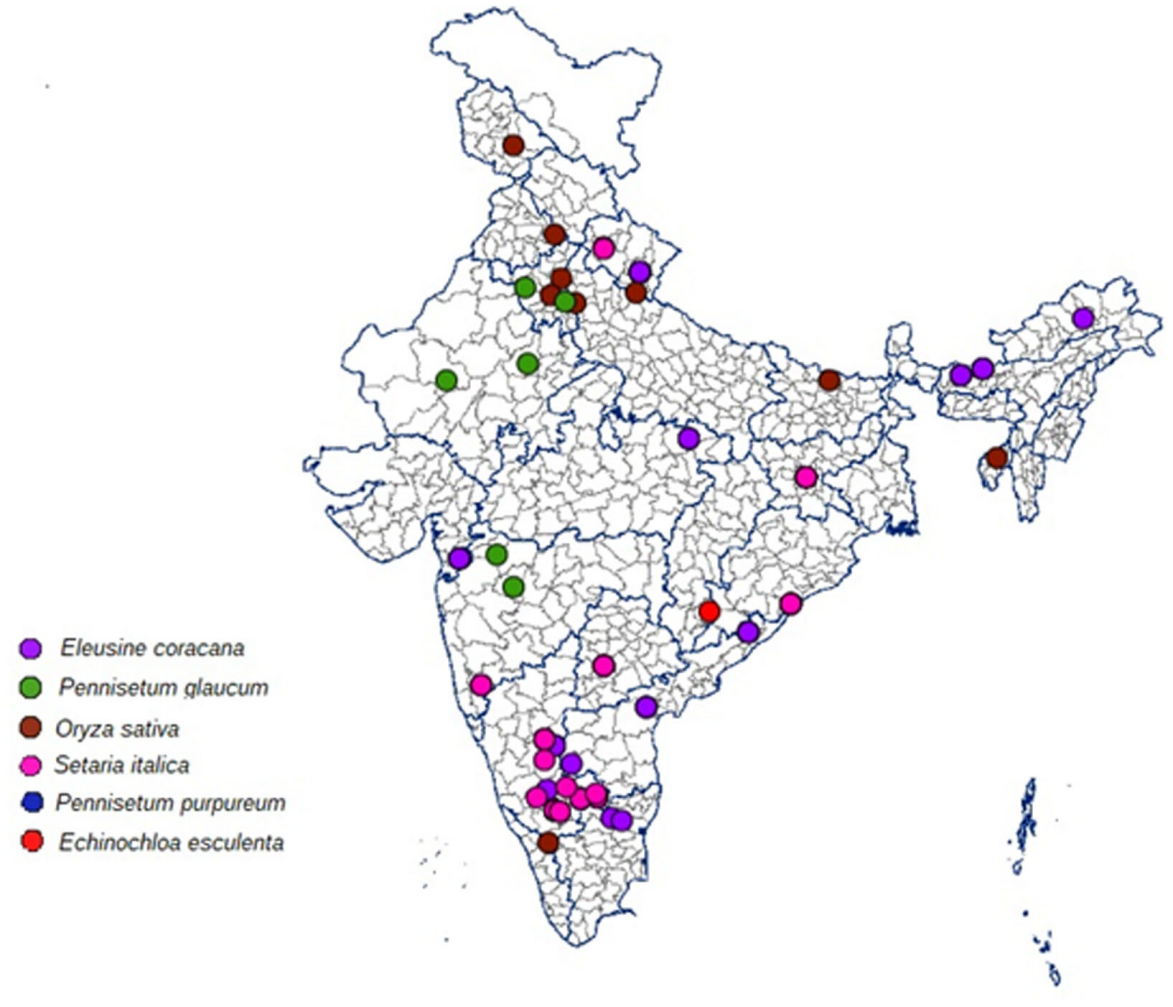
Details of geographical locations *Magnaporthe* isolates collected and isolated from major millets growing states of India.

The blast infected samples (leaf, neck and finger blast) from millet crops such as finger millet, barnyard millet, foxtail millet and pearl millet, and rice were collected from major millet growing areas in India including Bengaluru, Mandya, Hassan, Chitrdurga, Bangarpet, Ballari, Tumakuru and Kolar in Karnataka state; Perumallapalli and vizianagaram in Andhra Pradesh state; Waghai in Gujarat state; Athiyandal and Gudalur in Tamil Nadu state; Almora, Ranichauri, Udhamsingh Nagar and Gaja in Uttarakhand state; Jagdalpur in Chhattisgarh state; Ranchi in Jharkhand state; Rewa in Madhya Pradesh state; Dhule, Aurangabad and Kolhapur in Maharashtra state; Mandure and Durgapur in Rajasthan state; Hissar and Rohtak in Haryana state; Ghaziabad in Uttar Pradesh state; Berhampur in Odisha state, Palashquri, Jakappur, Gossaigaon and Rargiaghota in Assam state, Goba in Arunachal Pradesh; Kulai in Tripura and Hyderabad in Telangana; Khundwani in Kashmir; Madhubani in Bihar; Mohali in Punjab (**Table 1**; **Figure 1**). The samples were stored at -20 °C and used for fungal isolation in the later steps.

**Table 1:** Details of sample collection area from different crops across different growing regions of India.

S No	Crop	Location and state
**1**	Finger millet	Southern Karnataka, Perumal palli (AP), Athiyandal (TN), Jagadalpur (CH), Waghai (Gujarath), Odhissa, Rewa (MP), Ranchi, Jharkhand, Rajendranagar (Telangana)
**2**	Foxtail millet	Karnatka, Vzm (AP), Kolhapur Maharashtra, Jag (Chatisgarh), Rajendranagar (Telangana)
**3**	Pearl millet	Jodhpur & Jaipur (Rajasthan),Dhule & Aurangabad (Maharastra), Hissar (Haryana)
**4**	Barnyard millet	Jagdalpur (Chattisgarh) & Mandya (Karnataka)
**5**	Napier Bajra Hybrid	Bengalore (Karnataka)
**5**	Rice	Panipat & Rohtak(Haryana), Gazhiabad (U P), Khundwani (Kashmir), Mandya (KA), Madhubani(BH), Mohali (Punjab) , UdhamSingh Nagar(UK), Kulai (Tripura) and Gudalur (TN)

### Isolation and establishment of pure cultures

Two hundred blast-infected samples of millets (finger millet, barnyard millet, foxtail millet, and pearl millet), rice, and napier bajra hybrid grass were collected from various millet-growing districts in India. One hundred and thirty-six mono-sporidial isolates were established from 200 blast-infected samples using an efficient spore drop technique (Rajashekara et al., 2016), and single spore pure cultures were used for studying their morphology, molecular assays, and cultural aspects. These pure cultures were also preserved for future work in filter paper disc at -20 °C.

### Identification of *Magnaporthe* isolates

The established pure isolates of pathogen were identified based on its mycelial and conidial characteristics by comparing with standard descriptions of *Magnaporthe* species given by Saccardo (1880) and Hebert (1971).

### Studies on morphological and cultural parameters of pathogen

Morphological and cultural characteristics including growth characters, colony texture, surface topography and pigmentation etc. were studied on rice straw extract agar medium (rice straw-100 g, agar-20 g, agar, sucrose-20 g and water-1000 ml). For conidial characteristics for micro morphological traits, such as shape, length, and width, a minimum of twenty conidia per isolate were taken into consideration.

### Fungal genomic DNA isolation and PCR confirmation

The genomic DNA from blast isolates (136) was extracted by adopting CTAB (cetyl trimethyl ammonium bromide) method (Murray and Thompson, 1980). The quality and quantity of isolated genomic DNA of blast isolates were determined by spectrophotometer (Nanodrop ND 1000). After quantification, the isolated DNA samples were diluted to a final concentration of 25 ng/µl using nuclease-free water and used for PCR amplification. For confirmation of blast isolates, Internal Transcribed Spacer (ITS) region amplification [ITS-5: 5’-GGAAGTAAAAGTCGTAACAAGG-3’ and ITS-4: 5’-TCCTCCGCTTATTGATATGC-3’] was used in the study (White et al., 1990). PCR reactions were performed with a total volume of 20 µl reaction mix containing 5 ng of template DNA (2 µl), 1 pmol/µl each of forward (1 µl) and reverse primer (1µl), Taq^®^ enzyme and Takara Master Mix containing 25 mM MgCl2, 2 mM of each dNTPs, 1X Taq buffer(10µl), and nuclease free water (6µl). The thermal cycling program was: initial denaturation at 94 °C for 5 minutes followed by 35 cycles of denaturation for 45 seconds at 94°C, primer annealing for 45 seconds at appropriate temperature (**Table 2**), extension for 1 minute at 72 °C, and final extension for 5 minutes at 72 °C. The PCR amplicons were resolved using gel electrophoresis using 1.4% agarose in 1X TBE (Tris Borate EDTA) buffer. For electrophoresis we used 65 V for 1.5 h. The gel was documented using gel documentation unit by exposing to UV light.

**Table 2:** Details of PCR primers specific for virulence and avirulence genes and their sequences.

S No	Primer	Sequence (5’-3’)	Virulent/AVR gene	Annealing temp (°C)	Amplicon size (bp)	Reference
Virulence Gene						
1	ERG2	F: GCAGGGCTCATTCTTTTCTA R: CCGACTGGAAGGTTTCTTTA	Encodes for cutinase enzyme functioning in the degradation process of plant cuticle layer	60	1440	Soubabere et al., 2001
2	Cut1	F: TATAGCGTTGACCTTGTGGA R: TAAGCATCTCAGACCGAACC	Gene coding functioning in encoding secondary metabolites in antifungal target fungi on plant cell	60	800-1730	Soubabere et al., 2001
3	PWL/ PWL 2	F: TCCGCCACTTTTCTCATTCC R: GCCCTCTTCTCGCTGTTCAC	Is an avirulent gene and is host specific	60	800-900	Soubabere et al., 2001
4	MPG1	F; TCCCGGATCGTGGATAAATA R:ATTGTGTGCCTAGCCATTC	Hydrophobin-like protein	54	809	Sheoran et al., 2021
5	Mps1	CCAGACCGCAAAGTTCTCTC AGCAGAGTCCATGAGCGATT	TTK protein kinase	56	818	Sheoran et al., 2021
6	Slp1	TTCACCAAGACCAACCACAA AGTCGTGGTATGAGGCCAAC	WD repeat-containing protein slp1	56	790	Sheoran et al., 2021
7	Exo70	GCGAGCTCATAGAGGACACC AAAGTCCGCTCTTGGAGACA	Exocyst complex protein	56	960	Sheoran et al., 2021
8	ABC1	CTTTGAAGAGAAGCCCATCG CTTGACGCTCTTGGTGAACA	ATP-binding cassette ABC1 protein	54	958	Sheoran et al., 2021
9	Mlc	GACTCTCAGGCTTCCACCAA GGTGTCGACAGACTTGAGCA	Myosin regulatory light chain cdc4	57	487	Sheoran et al., 2021
10	Tps1	AGCCTTCTCGGGTAGCTAGG TGGTCGGACATGGACTTACA	Alpha, alpha-trehalose-phosphate synthase 1	57	950	Sheoran et al., 2021
Avirulence Gene						
1	Z41/Z42	TGCAGGCCCAAATCCGTAGGAA ACTGTCCGCCGCTCGTTTGG	*Avr-Pii*	55	508	Lopez et al., 2019
2	Z23/Z24	TCCAATTTATTCAACTGCCACTC GTAAACCTCGTCAAACCTCCCTA	*Avr-Pik*	55	532	Lopez et al., 2019
3	Z21/Z22	AATCCCGTCACTTTCATTCTCCA GTCGCAAGCCTCGTACTACCTTT	*Avr-Pizt*	55	637	Lopez et al., 2019
4	Z27/28	CCCATTATCTTACCAGTCGCTTGA ATTCCTCCCGTAAACAGTAAACC	*Avr-Pia*	55	868	Lopez et al., 2019

### Sequencing and phylogenetic analysis

The PCR amplified DNA fragments were sequenced through outsourcing (Chromous Biotech Pvt. Ltd., Bengaluru), and used for sequence alignment against *Magnaporthe* genome sequences using blast search tool at NCBI (National Centre for Biotechnology Information: https://blast.ncbi.nlm.nih.gov/Blast.cgi). The sequence reads were then used for phylogenetic analysis. Also, the ITS DNA sequences of all 136 isolates were deposited in NCBI GenBank (http://www.ncbi.nlm.nih.gov), for accession numbers.

### PCR assay of *Magnaporthe* isolates for virulence and avirulence genes

The *Magnaporthe* isolates genomic DNA was extracted from the single spore pure cultures of fungal mycelia. DNA fragment was amplified using the primers sets specific to virulent and avirulent genes. We studied ten virulence (*vir*) (*ERG2*, *Cut1*, *PWL2*, *MPG1*, *MPS1*, *Slp1*, *Exo70*, *ABC1*, *Mlc* and *Tps1*) and four avirulen ce(*Avr*) (*Avr-Pii, Avr-Pik, Avr-Pizt* and *Avr-Pia*) genes (**Table 2**). PCR reactions were performed with a total volume of 20 µl reaction mix containing 5 ng of template DNA (2 µl), 1 pmol/µl each of forward (1 µl) and reverse primer (1µl), Taq^®^ enzyme and Takara Master Mix containing 25 mM MgCl2, 2 mM of each dNTPs, 1X Taq buffer (10µl), and nuclease free water (6µl). The thermal cycling program was: initial denaturation at 94 °C for 5 minutes followed by 35 cycles of denaturation for 45 seconds at 94°C, primer annealing for 45 seconds at appropriate temperature (**Table 2**), extension for 1 minute at 72 °C, and final extension for 5 minutes at 72 °C. PCR amplicons were resolved in 1.5% (w/v) agarose gel through electrophoresis. The PCR amplicons were recorded using binary code of 1 and 0 for presence and absence, respectively.

### Statistical analysis

The binary data of PCR-based amplicons was used for genetic diversity analysis of 136 blast isolates for 10 virulent and 4 avirulent genes. Using Power Marker version 3.25, the polymorphic information content (PIC), gene diversity, and the major allele frequency were calculated (Liu and Muse, 2005). In the GenAlEx v. 6.502 software, pairwise “F” statistics (FST) and Nei’s genetic distance (Nei, 1987) were computed (Peakall and Smouse, 2012).

### Analysis of population structure and AMOVA

The STRUCTURE (v. 2.3.4) software was used to analyze the population structure and familial ties of 136 blast isolates in relation to the target virulent and avirulent genes (Pritchard et al., 2000). The actual number of subpopulations, designated as K value was determined by admixture ancestry model with *ad hoc* statistic ΔK. The run length given was 50,000 burning period length followed by 50,0000 Markov Chain Monte Carlo (MCMC) replications. Further each K value was run 10 times with K investigated from 1 to 10. The true number of K was further identified using the STRUCTURE harvester (Earl, 2012) and the best K value was determined on the basis of LnP(D) and Evanno’s ΔK proposed by Evanno et al. (2005). Further, the number of subpopulations with the isolates was confirmed by drawing a cladogram using interactive tree of life (iTOL v6.6) online tool (https://itol.embl.de/). The molecular variation between subpopulations was examined and reported as Analysis of molecular variance (AMOVA) by using the GenAlEx version 6.502 software (Peakal and Smouse, 2006).

Furthermore, by performing principal component analysis (PCA) with the help of the R software’s “factoextra” package, the virulent genes of isolates were categorized according to host plants, tissues affected, and the site of collection (Kassambara and Mundt, 2017). The contribution of each pathogenic gene to the overall diversity of *Magnaporthe* was also evaluated by PCA analysis.

## Results

### Collection samples and establishment of blast isolates

One hundred and thirty-six pure blast pathogen isolates from finger millet, foxtail millet, barnyard millet, pearl millet, rice and napier bajra hybrid were established from the blast infected samples collected from major millet growing regions of India. Among these 136 blast isolates, 78 were from finger millet, 36 from foxtail millet, four from barnyard millet, seven from pearl millet, ten from rice, and one isolate from napier bajra hybrid.

### Cultural and morphological characterization

**Figure 2:**
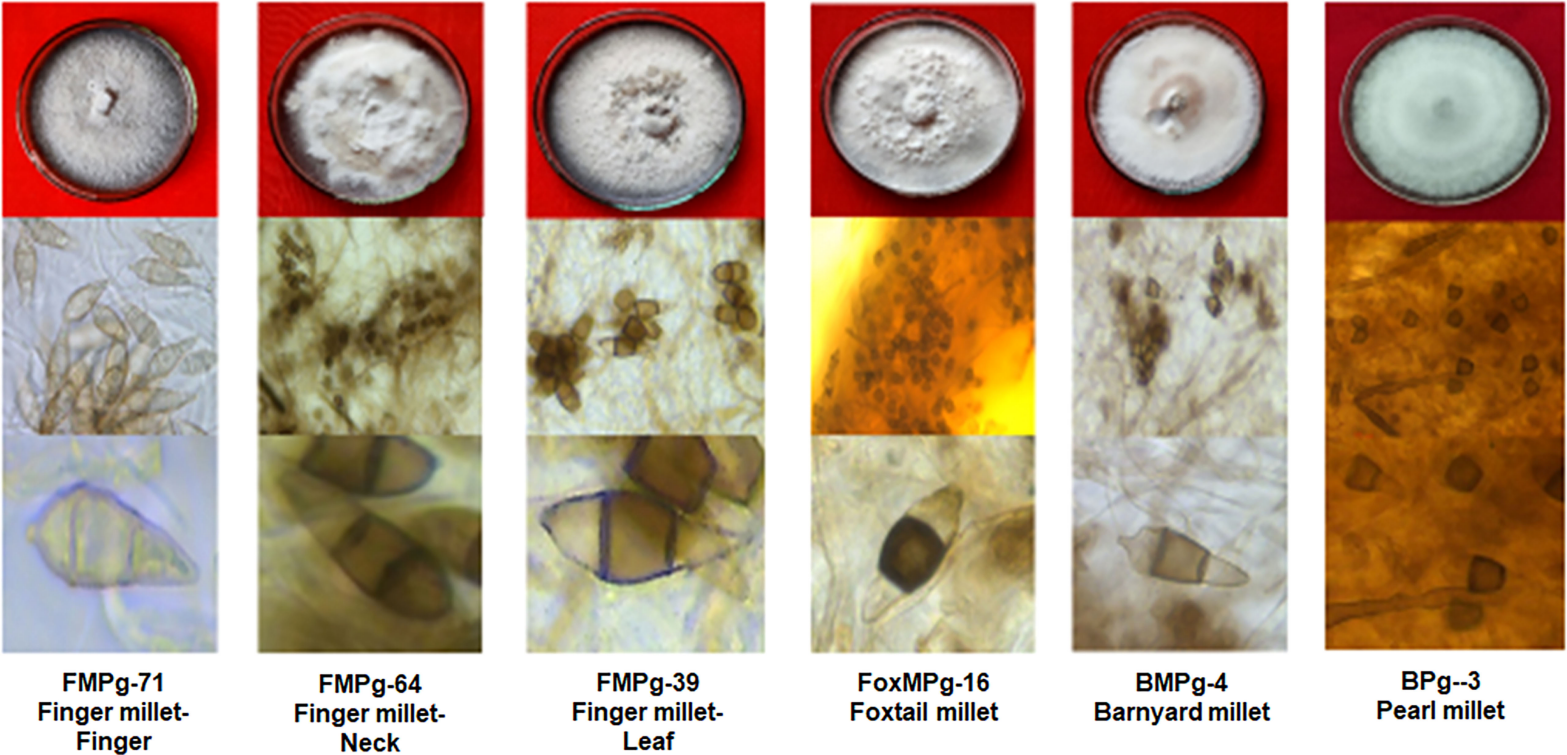
Representative pictures of blast isolates from different millet crops and their conidial morphology.

Regardless of the crop or the geographical location of samples collected, variations were seen in the different isolates with respect to colony characters, pigmentation, and micromorphological characteristics. Cultural and morphological factors were also evaluated. White, whitish grey, creamish grey, and grey are some of the colors recorded (**Figure 2**). Regardless of the type of crop and location, the melanin pigment, indicated by the colony pigmentation, varied across the different isolates. Microscopic observation of conidia based on their length and shape divided them into three classes: short pyriform shape (25 μm), pyriform shape (25–30 μm), and long pyriform shape (>30μm). Conidia sizes in finger millet isolates 17.50×7.94 μm (FMPg 71) to 27.83×9.50 μm (FMPg 72) in representative isolates whereas foxtail millet isolates accounted 21.65×8.87 μm (FoxM Ps15) to 22.13×9.32 μm (FoxM Ps16) and in barnyard millet it ranged from 22.12×9.25 μm (BMPg1) to 23.41×9.15 μm (BMPg4). Similar variation was seen in isolates of pearl millet and rice (**Table 3**). The pathogen identification based on culture morphologyy and micromorphological characteristics was done following the standard descriptions of the *Magnaporthe* species provided by Saccardo (1880) and Hebert (1971).

**Table 3:** Cultural and morphological characteristics of representing *Pyricularia* isolates across the geographical locations of India.

Sl.No	Isolate	Crop	Plant Tissue	Macro morphological characters						Micro morphological characters		
				Colony character	Radial growth (mm/cm)*	Days to cover full plate	Melanin pigment	Topography	Colony margin	Conidial shape	Conidial size (µm)	
											Range	Average
1	FMPg41	Finger millet	Leaf	Whitish grey	55.5	18	++	Raised	Regular	Medium pyriform conidia	24.9 -31.06×8.30-10.55	22.9×9.04
2	FMPg72	Finger millet	Leaf	Whitish	74.8	16	++	Raised	Irregular	Long pyriform conidia	25.04-29.05× .98-10.02	27.83×9.50
3	FMPg64	Finger millet	Neck	Grey	71	15	+++	Flat	Regular	Medium pyriform conidia	20.86-24.01×7.78-9.2	24.01×8.43
4	FMPg66	Finger millet	Neck	Greyish white	85.7	13	++	Raised	Irregular	Medium pyriform conidia	20.56-26.88×6.85-7.99	24.68×7.50
5	FMPg70	Finger millet	Finger	Whitish	66.8	15	++	Slightly raised	Regular	Medium pyriform conidia	20.88-27.56×8.12-9.86	23.54×9.28
6	FMPg71	Finger millet	Finger	Whitish grey	82.8	13	++	Raised	Irregular	Short pyriform conidia	16.16-19.62×7.13-8.14	17.50×7.94
7	FoxM Ps15	Foxtail millet	Leaf	Creamy white	82	13	++	Raised	Regular	Medium pyriform conidia	15.91-24.27×7.9-9.62	21.65×8.87
8	FoxM Ps16	Foxtail millet	Leaf	Grey	88	12	+++	Flat	Regular	Medium pyriform conidia	17.75-27.80×8.32-10.23	22.13×9.32
9	BMPg1	Barnyard millet	Leaf	Whitish grey	71	20	++	Flat	Irregular	Medium pyriform conidia	20.2-26.79×8.39-10.18	22.12×9.25
10	BMPg4	Barnyard millet	Leaf	Whitish grey	72	13	+++	Raised	Regular	Medium pyriform conidia	21.2-27.69×8.39-10.18	23.41×9.15
11	BPg3	Pearl millet	Leaf	Greyish white	73	12	+++	Slightly raised	Regular	Long pyriform conidia	25.01-28.14×6.99-8.97	25.9×7.9
12	BPg6	Pearl millet	Leaf	Grey	71	14	+++	Flat	Regular	Medium pyriform conidia	19.39-25.47×8.07-9.63	22.9×9.04
13	RPo2	Rice	Leaf	Whitish grey	74.5	13	+	Flat	Regular	Medium pyriform conidia	20.47-24.19×8.69-11.26	21.12×9.55
14	RPo3	Rice	Leaf	Grey	71.8	12	+++	Raised	Regular	Medium pyriform conidia	19.45-25.26×8.15-10.34	20.34×9.14

Melanin pigment: - No pigmentation, + Pigmentation in < 20 % of the mycelia area, ++ 20-50 %, +++ 50-80%, ++++ > 80 %; Sporulation.

### PCR based molecular characterization of *Magnaporthe* isolates

The universal primers ITS5 and ITS4 amplified a 600 bp DNA fragment. The PCR amplification was then purified and sequenced. The high-quality gene sequences were then submitted to NCBI and were given GenBank accession numbers (**Supplementary Table 1**). All the isolates showed maximum matching similarity to *Magnaporthe* spp. in NCBI database and confirming purely a blast pathogen infecting different cereal hosts.

### Analysis of *virulent* genes in blast isolates

**Figure 3:**
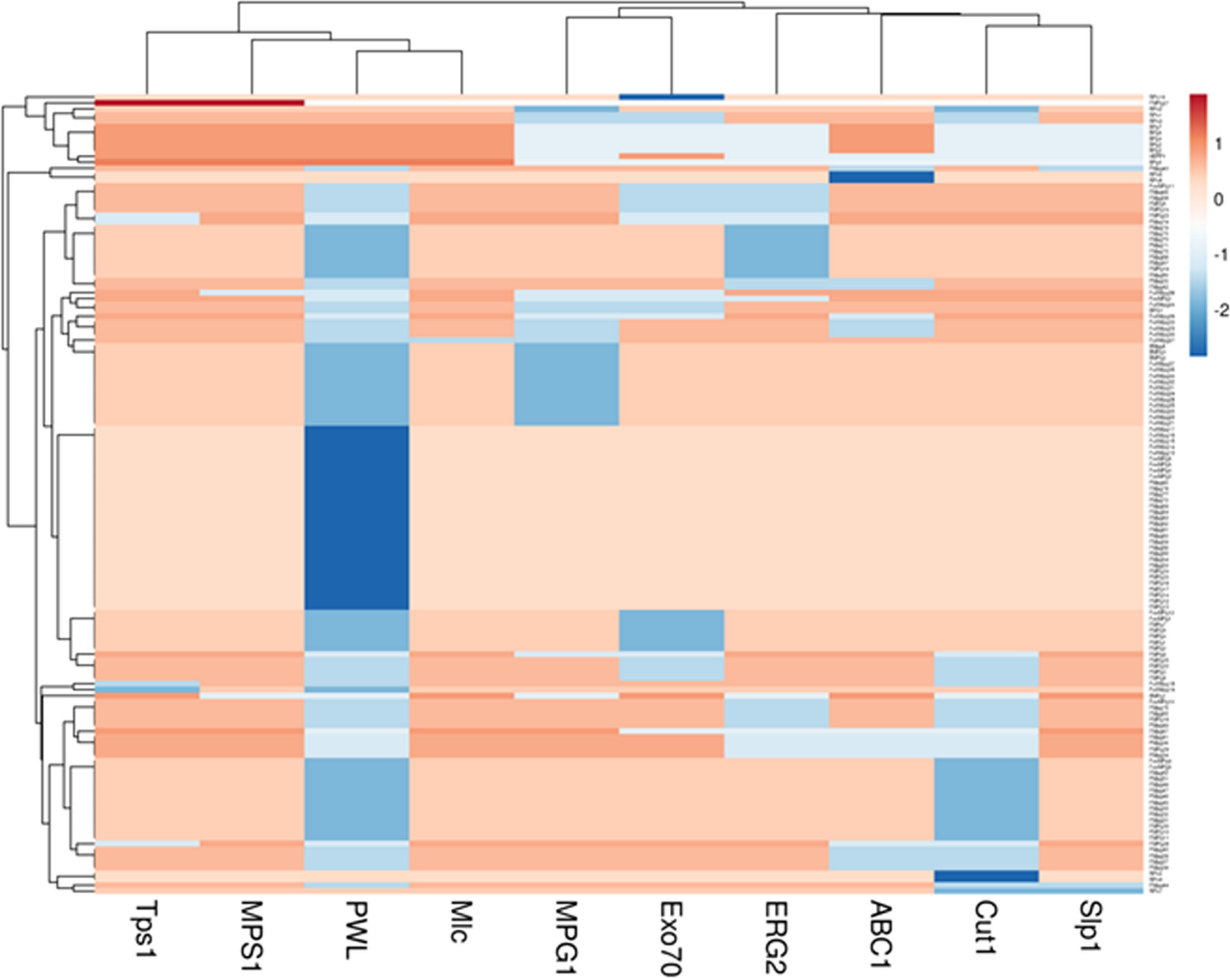
Heat map for virulence genes of *Magnaporthe* infecting millets.

Virulent genes such as *ERG2, Cut1, PWL2, MPG1, MPS1, Slp1, Exo70, ABC1, Mlc*, and *Tps1* were PCR amplified. These genes encode for enzymes, proteins, and secondary metabolites having role in pathogenicity/virulence. Among them, *MPS1* and *Mlc* were amplified in 134 of the 136 isolates (98.53%) with corresponding band sizes of 809 bp and 487 bp, respectively. *Tps1* (950 bp) and *Slp1* (750 bp), were amplified in 131 (96.33%) and 125 (91.92%) isolates, respectively (**Supplementary Figure 1**). Only 17 isolates or 12.5% of them were found positive for *PWL* with DNA amplicons in the range of 800-900 bp. The *Cut1* (800-1730bp) marker could amplify in 85 isolates (62.5%) and *ERG 2* (1440 bp) marker was amplified in 98 isolates (72.05%). The presence of *MPG1* (809 bp), *Exo70* (960 bp), and *ABC1* (958 bp) in 100, 101, and 114 isolates, respectively, accounted for 73.53, 74.26, and 83.83 percent (**Table 4**; **Figure 3**).

**Table 4:** Distribution of virulence and avirulence genes in blast fungus isolated from millets and rice.

Sl.No.	Virulent/AVR gene	Proportion of virulence and avirulence gene (%) *					
		Finger millet	Foxtail millet	Pearl millet	Barnyard millet	Rice	Fodder grass (Napier bajra hybrid)
Virulence genes							
1	ERG2	64.10	91.67	14.29	75.00	100.00	0.00
2	Cut1	57.69	88.89	14.29	75.00	40.00	0.00
3	PWL/PWL 2	0.00	0.00	85.71	0.00	100.00	100.00
4	MPG1	97.44	47.22	0.00	0.00	70.00	0.00
5	MPS1	100.00	97.22	100.00	75.00	100.00	100.00
6	Slp1	96.15	100.00	14.29	100.00	90.00	0.00
7	Exo70	76.92	80.56	0.00	100.00	70.00	100.00
8	ABC1	82.05	88.89	85.71	100.00	80.00	0.00
9	Mlc	98.72	97.22	100.00	100.00	100.00	100.00
10	Tps1	96.15	94.44	100.00	100.00	100.00	100.00
Avirulence genes							
1	Z41/Z42 *(Avr-Pii)*	100.00	11.11	14.29	100.00	0.00	0.00
2	Z23/Z24 *(Avr-Pik)*	0.00	0.00	42.86	0.00	50.00	100.00
3	Z21/Z22 *(Avr-Pizt)*	98.72	100.00	14.29	100.00	100.00	0.00
4	Z27/28 *(Avr-Pia)*	88.46	100.00	100.00	50.00	80.00	100.00

*The number of isolates used for PCR assay, Finger millet (n = 78), Foxtail millet (n = 36), Pearl millet (n = 7), barnyard millet (n = 4), Rice (n = 10) and Napier bajra hybrid=fodder (n = 1).

### Avirulent genes in blast isolates

*Avr-Pizt*, one of the four *AVR* genes examined, was found in the majority (94.12%) of the isolates of finger millet, foxtail millet, barnyard millet, and rice but was absent from all but one isolate of pearl millet (BPg1). The other *Avr* genes have frequencies of 91.92% (*Avr-Pia*), 63.97% (*Avr-Pii*), and 6.62%. (*Avr-Pik*). *Avr-Pik* was only detected in the rice and pearl millet isolates, While *Avr-Pik* was solely found in rice and pearl millet isolates, *Avr-Pii* was found in the majority of isolates of finger millet and pearl millet isolates (**Table 4**; **Supplementary Figures 2,** **3**).

### Gene distribution and diversity

**Figure 4:**
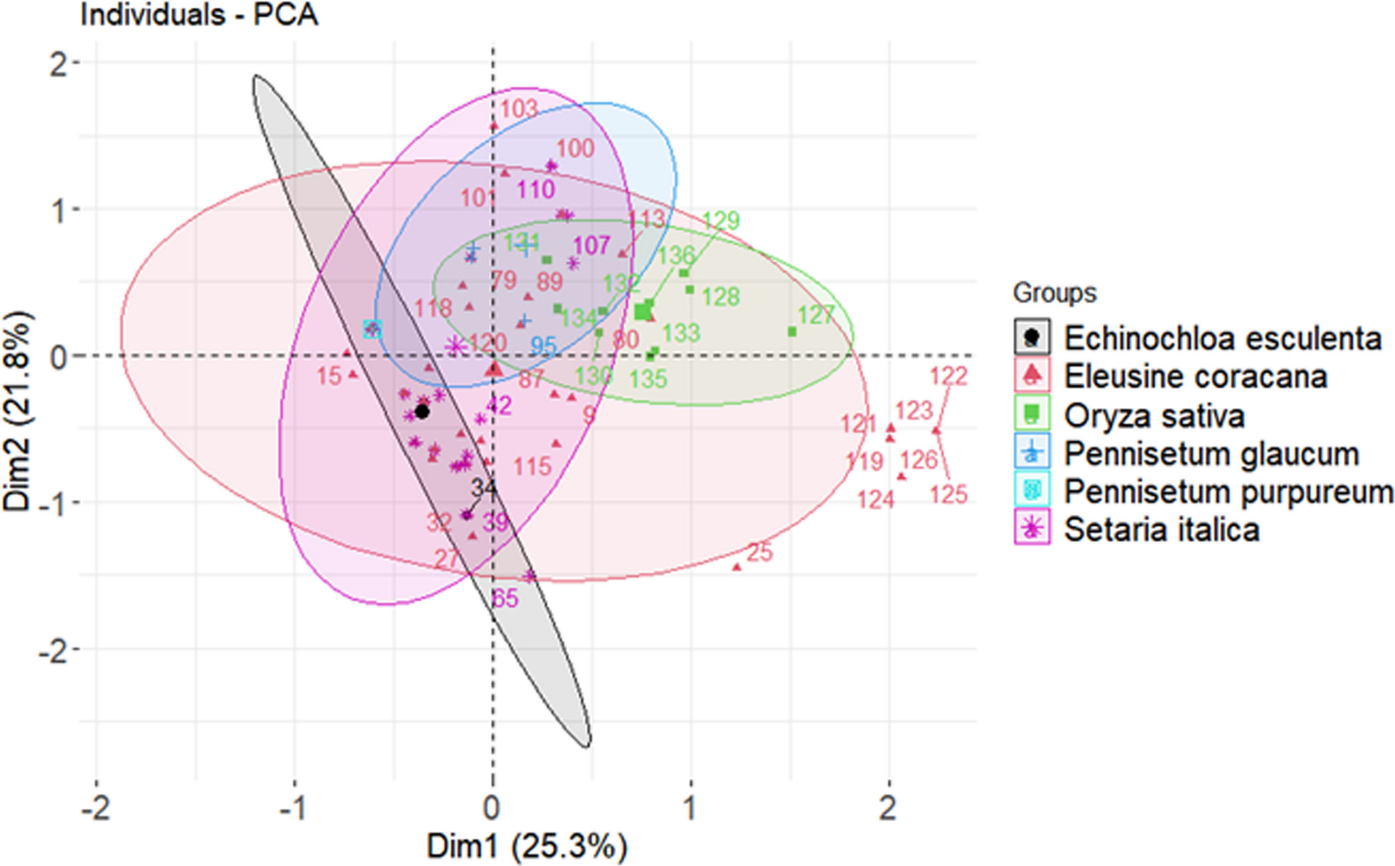
Crop wise virulence genes distribution of *Magnaporthe* infecting millets.

**Figure 5:**
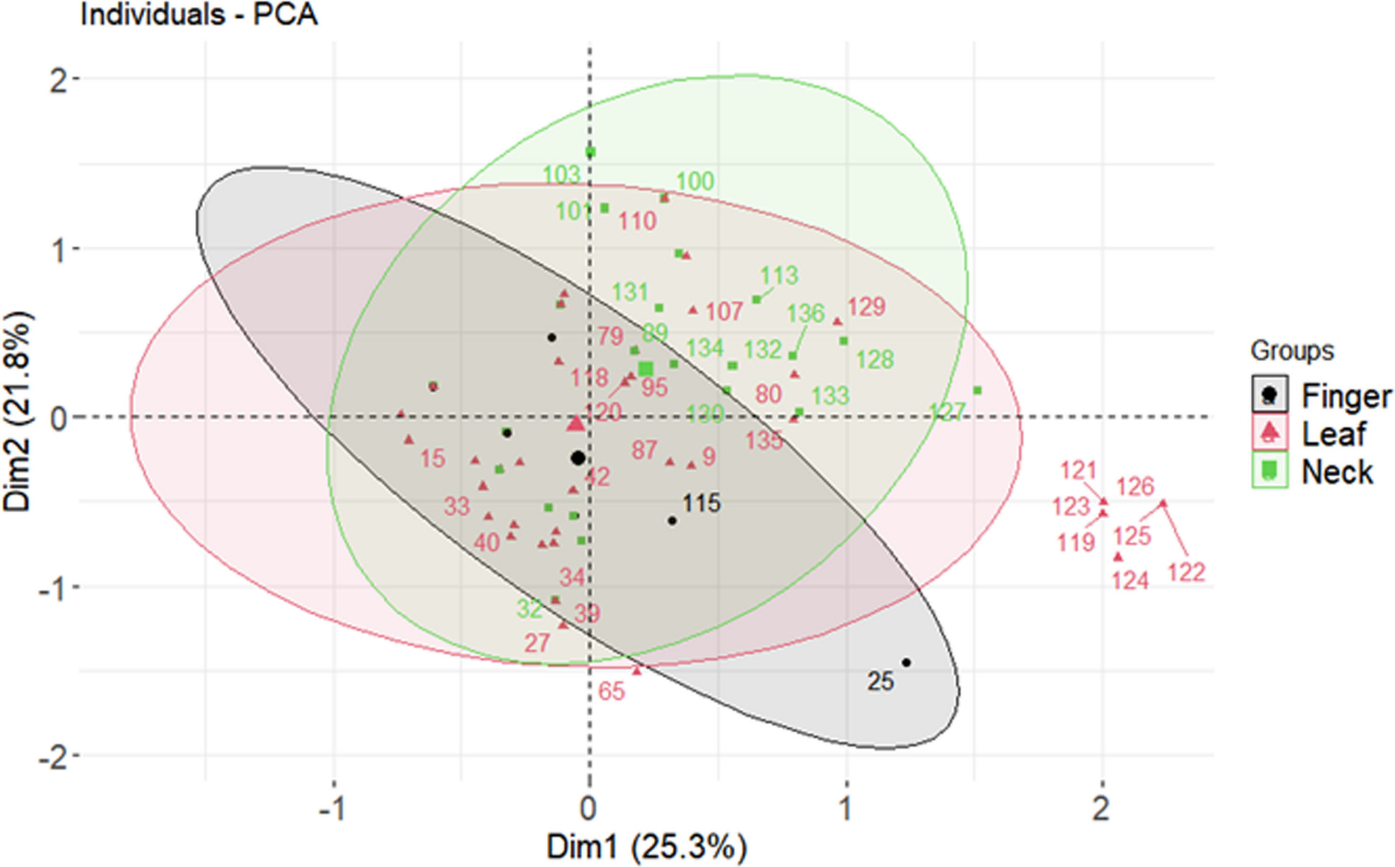
Tissue wise virulence genes distribution of *Magnaporthe* infecting millets.

Nearly 50% of the genetic diversity of the *Magnaporthe* population was described by the first two components of the principal component analysis (**Supplementary Figure 4**). It was evaluated for how pathogenicity genes are distributed among the *Magnaporthe* isolates from different host species. Since many of the virulent genes were found in isolates from several host plants, there was no distinct pattern of distribution of these genes in different host plants (**Figure 4**). There was no distinct categorization of isolates based on gene distribution with respect to the tissue of infection as well (**Figure 5**). A similar pattern was seen when categorizing these isolates by their geographic location (**Supplementary Figure 5**).

### Population structure analysis

**Figure 6:**
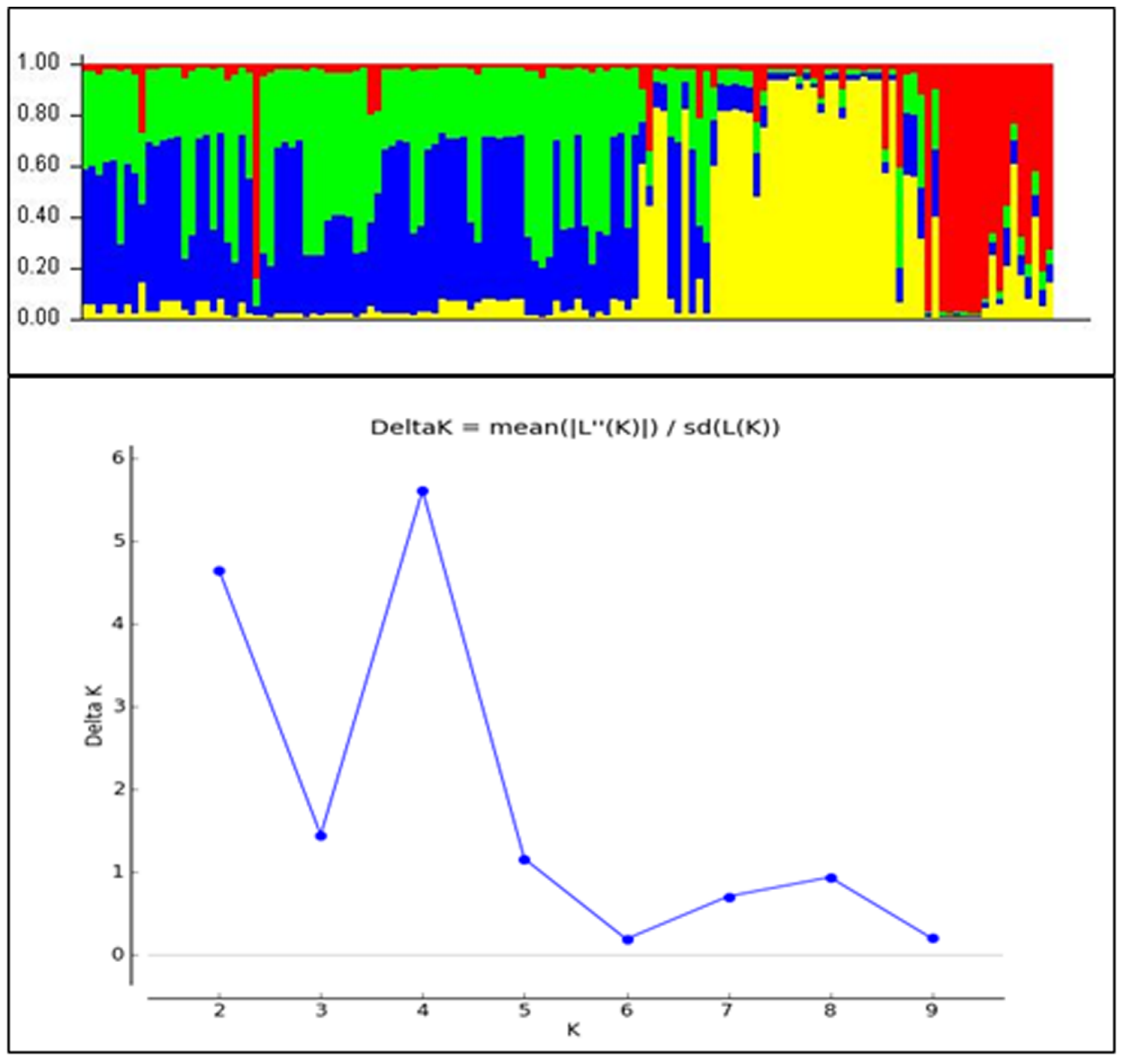
Population clustering of *Magnaporthe* isolates infecting millets at estimated membership fraction for K = 4.

**Figure 7:**
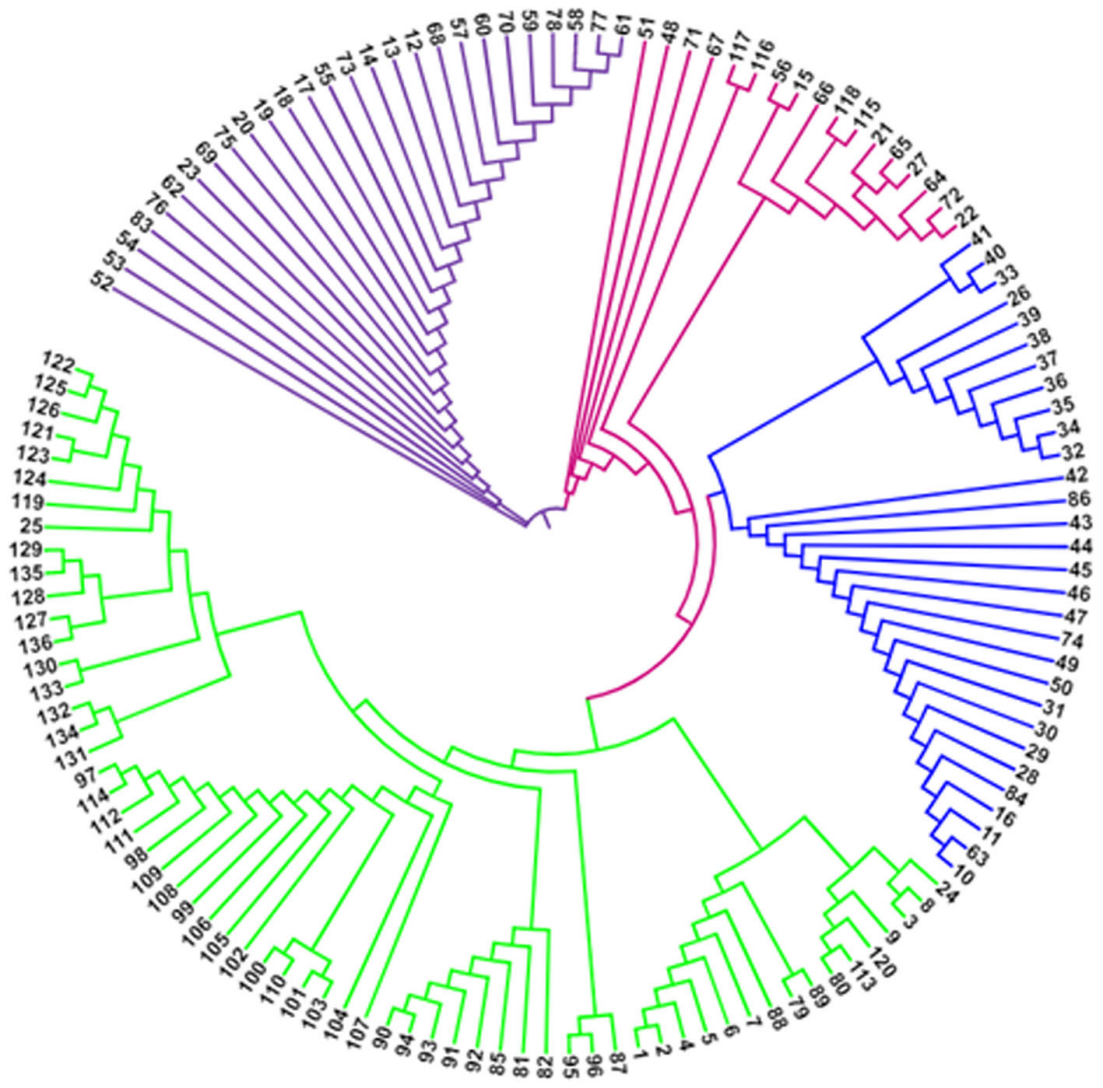
Cladogram of *Magnaporthe* population infecting millets.

The classification of *Magnaporthe* populations into four subpopulations based on their family links was done using population structure analysis by utilizing gene-based functional markers data of 136 *Magnaporthe* isolates and was also validated using K analysis (**Figure 6**). These 136 blast isolates from various locations of India were shown to contain four subpopulations. Additionally, the cladogram generated using these molecular data also indicated the existence of four subpopulations within the original population of 136 isolates (**Figure 7**).

### AMOVA analysis

**Figure 8:**
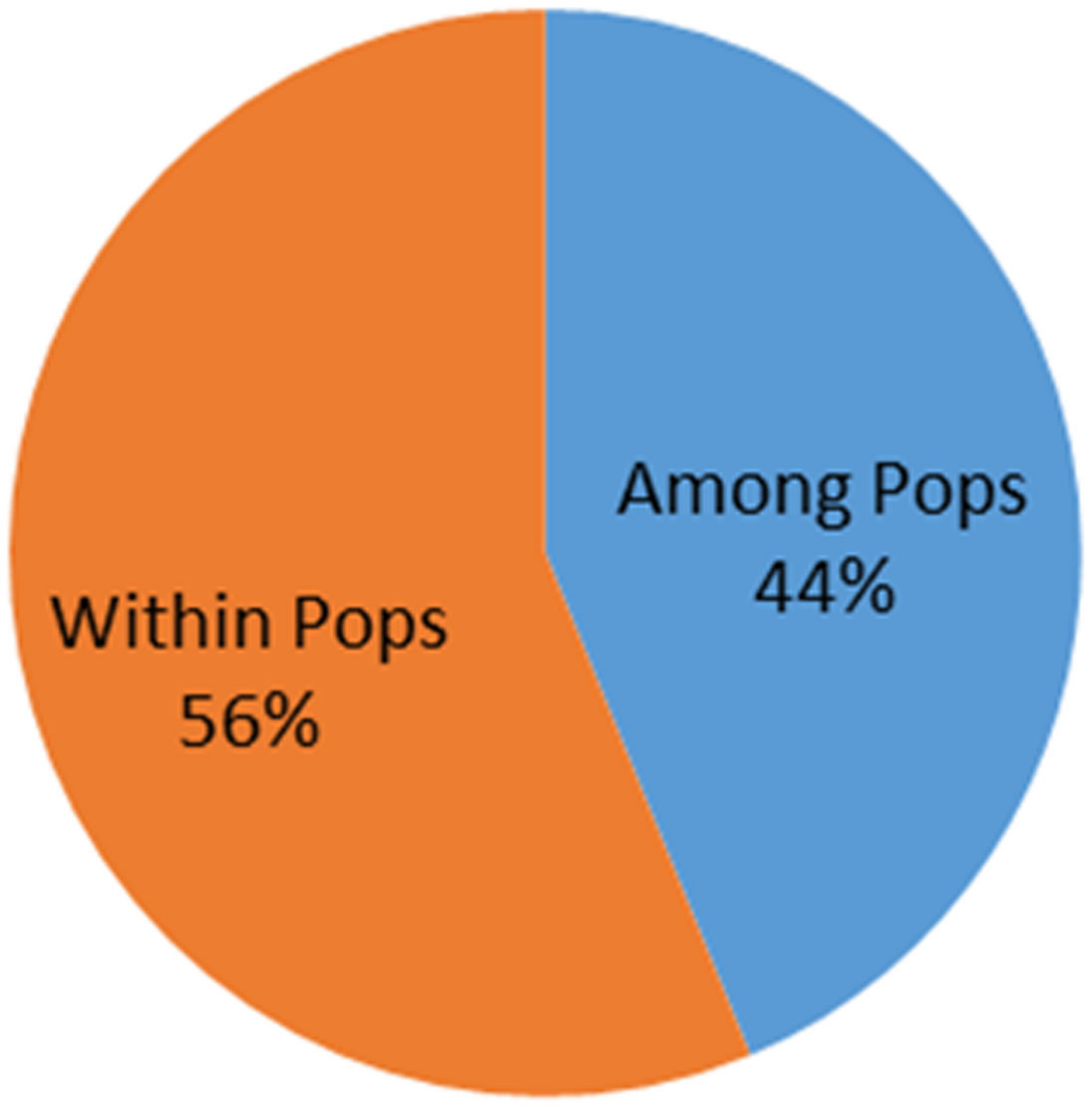
Percentage of molecular variance within and among the *Magnaporthe* population infecting millets.

AMOVA analysis was used to evaluate the existing genetic diversity both within and between the 136 *Magnaporthe* isolates. The results indicated presence of more molecular variation within-population (56%) of isolates infecting various millet crops, rice, and napier hybrid bajra than the among the population (44%) (**Figure 8**).

## Discussion

For a long time, plant pathologists and crop improvement scientist especially plant breeders have tried to understood how important is the pathogen variance in terms of efficacy and endurance of host plant resistance (HPR). The virulent pathogen may interact with a particular host genotype, which could result in the “breakdown” of resistance extremely quickly (Brown, 1995). The identification of diversity with respect to virulence variation (races) within the pathogen population has traditionally been used to detect pathogen variation. To do this, a set of pathogen isolates were inoculated onto a seedlings of different host plants with specific resistance genes (host differentials), and the disease reactivity (resistant or susceptible) for each isolate was recorded visually. The development and application of host resistance has greatly benefited from this method of pathogen population monitoring (Roelfs, 1985; Wolfe and Limpert., 1987; Andrivon and De Vallavieille-Pope, 1993). It has also provides an insight into the evolution of prevailing *Magnaporthe* populations in response to host resistance (*R*) gene selection (Kolmer, 1989; Andrivon and De Vallavieille-Pope, 1993; Wolfe and McDermott, 1994). In many pathosystems today, pathotype monitoring is still widely utilized to convey on-time information about the makeup of blast pathogen populations which is very much essential for breeding initiatives and the deployment of resistance crop plant genotypes.

An understanding of the composition, host jump and distribution of *Magnaporthe* populations in small millet hosts and their geographical locations across India is important in highlighting our knowledge of the biology of the pathogen and potentially adaptive ability and genotypic diversity in these pathogen population. In this study, the analysis of morphogenesis could capture various growth characteristics of *Magnaporthe* sp. infecting pearl millet, finger millet, foxtail millet, barnyard millet, and rice plants.

The current study evaluated the cultural- morphological and molecular characterization, and diversity of virulent and avirulent genes in the *Magnaporthe* population from small millets, rice, and napier hybrid bajra hosts representing geographical locations across India. This is a prerequisite to understand the distribution and variation of pathogenicity determinant genes across the geographical locations in various millets, and this information is currently lacking. The knowledge gained through this study from these populations will be very beneficial for understanding pathotypes and developing and deployment of disease-resistant millets cultivars.

Due to the overlapping cultural and morphological characteristics, we employed universal DNA marker (ITS) sequencing to precisely identify blast pathogens. Previous studies relied on the importance of ITS markers in characterization of *Pyricularia* isolates (Hirata et al., 2007; Abed-Ashtiani et al., 2016). ITS gene sequence could identify blast pathogen *Pyricularia* at the species level (Hirata et al., 2007). In this study, using the ITS specific ITS4 and ITS5 primers we could confirm that all the test pathogen isolates employed in the present research belonged to the *Magnaporthe* spp.

During the process of plant infection, pathogens secrete a set of molecules termed as effectors. These effectors are the key determinants of pathogen virulence. The products of plant resistance (*R*) gene play an important role in the detection and interaction with pathogen effects called avirulence (Avr) proteins and induce R-Avr mediated incompatible disease reaction (Sharma et al., 2012). In this study, out of the 10 investigated virulent genes, majority of 136 *Magnaporthe* isolates could amplified for *MPS1* (TTK Protein Kinase) and *Mlc* (Myosin Regulatory Light Chain edc4) genes. This amplification pattern was independent of the crop and location of sampling, suggesting these genes may have significant role virulence determination. Additionally, among *Avr-Pii, Avr-Pik, Avr-Pizt*, and *Avr-Pia* genes, the *Avr-Pizt* had the highest frequency of amplification followed by *Avr-Pia*. Interestingly, *Pizt* and *Pita* are among the most broad-spectrum blast resistance genes (Devanna et al., 2022). Also, it’s intriguing to note that the *Avr-Pik* gene was completely lacking in the blast isolates obtained from finger millet, foxtail millet, and barnyard millet while being present in the fewest isolates from the other three, indicating noticeably significant variation both across and within them.

Population structure of *Magnaporthe* isolates both within the same species and across geographic locations are probably related to variations in evolutionary history and ecology (Varshney et al., 2009). Similar findings were made by Tosa et al. (2007), who discovered that *Setaria* and *Oryza* isolates were genetically more similar and shared the avirulence genes *PWT1* and *PWT2*. The prediction of new races and their interactions in a regional agro-ecology may be made possible by using the information of the local pathotype diversity in *Pyricularia oryzae* and the virulence genes. The discovery of virulence genes may serve as a crucial foundation for the identification and generation of resistance genetic sources to rice blast (Dubina et al., 2020). In the current study, *Mlc* and *MPs1* were found in 98.53% of isolates, followed by *TPs1* (96.33%). Whereas, this frequency was 91.92% for *Slp1*, 83.83% for *ABC1*, 74.26% for *Xxo70*, 73.53% for *MPG1*, 72.05% for *ERG2*, 62.50% for *Cut1*, and 12.5% for *PWL*. While *PWL* was amplified only in isolates from pearl millet and rice, and was completely absent in finger millet, foxtail millet, and barnyard millet isolates, the *Mlc* and *MPs1* genes are dispersed regardless of the geographical location and host crop.

The lack of amplification of *Cut1, ERG 2, MPG1 Exo 70*, and *PWL* in most of the isolates can be attributed to evolving pattern of these genes. Both pathogenic virulence factors and a diversity of pathogen populations are caused by the mutation. The prevalence of transposon elements may contribute to the *P. oryzae* pathogen’s mutation (Kito et al., 2002; Singh et al., 2018).

The *Cut1* which codes for cutinase enzyme involved in the plant cuticle layers degradation process (Sweigard et al., 1992), whereas Pwl2, avirulent gene which is a host specific in nature (Valent and Chumley, 1994). Erg2 is an antifungal target for blast pathogen and also secretes secondary metabolite in plant cells (Keon et al., 1994). Reflinur et al. (2005) characterized 5 races of *P. oryzae* distributed among 230 isolates with virulence gene-coding specific markers and found eight haplotypes among the population studied. Li et al. (2010), *P. oryzae* isolates are genetically stable but have high pathogenic variations. According to Leung et al. (1993), host plant selection, is an evolutionary process that significantly influences the development of pathogen genetic variants. Leung et al. (2003) reported DNA fingerprint studies of *P. oryzae* strains and showed that deployment monogenic(one) variety has low genetic variation when compared to isolates of *P. oryzae* collected from areas planted with polygenic varieties. Among *Avr* genes, *Avr-Pizt* was more frequently (94.12%) amplified in *Magnaporthe* isolates of millets followed by *Avr-Pia* (91.92%). However, Lopez et al. (2019) reported that *Avr-Pik*, *Avr-Pita* and *Avr-Pii* were more frequently occurring genes with 81.50%, 64.16% and 47.98% frequency, respectively. Whereas, the frequency of *Avr-Pizt* and *Avr-Pia* was 19.08% and 5.20%, respectively. It was also laid out that the presence of *Avr* genes in *M. oryzae* is strongly associated with agro-ecosystems where the complementary resistant (*R*) genes exist.

Jia et al. (2012) studied the *Avr* genes from the blast isolates collected from the southern US during (1970 to 2009), and found that majority of the isolates had *Avr-Pita* (65.7%). The relative frequencies of other *Avr* genes ranged from 0.3% to 57.1%. Similarly, in Yunnan province of China, *AvR-Pii* was present in 82 out of 454 field isolates of *M. oryzae* studied (Lu et al., 2019). In Eastern India, Imam et al. (2015) reported that the occurrence *Avr-Pizt* and *Avr-Pik* were in highest frequency (100%), whereas *Avr1-CO39* was in the lowest (2%) frequency. In a similar study, the frequency of *PWL-2*, *Avr-Pii* and *Avr-Pizt* genes was 100%, 60% and 54%, respectively in Thailand (Sirisathaworn et al., 2017). These studies infer that there is a lot of diversity in the frequency of blast pathogen avirulence genes in millet isolates of India, as evidenced by *Avr- Pizt* and *Avr-Pia* having the highest frequency of 94.12% and 91.92%, respectively. Though *Avr-PWL3* is rarely present in *M. oryzae* isolates from rice (Kang et al., 1995), but in our study, its frequency is more than *Avr-Pia*.

The distribution of molecular variance in the population also indicates the diverse evolutionary history, both temporal and spatial. In the present study, we observed comparatively higher molecular variance within the population in comparison to among the population. This indicates that isolates within a given geographical regions have evolved more. This implies that there is more chance of evolution of new virulent strains from these populations, and this might affect the present *R*-Avr balance in these crop growing areas.

## Conclusions

Present study provides some novel insight into the genetics of adaptation of *Magnaporthe* populations to millet crops. The genetic diversity with respect to virulent and avirulent gene observed in the populations adapted to millet might show the pathogenic variations among the *Magnaporthe* populations. Thus, understanding the genetic structure and pathogenic nature of the *Magnaporthe* populations belonging to different clonal-lineages will help in designing the strategies for millet blast management programs especially by deployment of resistant genes for eco-friendly management of blast through host plant resistance.

## Data availability statement

The datasets presented in this study can be found in online repositories. The names of the repository/repositories and accession number(s) can be found in the article/**Supplementary Material**.

## Author contributions

KP, HV, and SP conceived the study, designed the experiments, and participated in drafting the manuscript. BJ, HRR, FK, CB, VU, TP, LR, MR, PS, PN, GR, ID, HP, AJ, SS, SN, GP, and TN collected the data and performed the analysis. HR, BD, SP, and CA performed the diversity analyses. KP, HR, BD, CA, and SP wrote the manuscript. KP, ID, TN, BD, and SN provided resources and improved the manuscript. All authors contributed to the article and approved the submitted version.
