# Supplementary material for: Exploring the diversity of virulence genes in the Magnaporthe population infecting millets and rice in India
Source: Front Plant Sci. 2023 May 9;14:1131315. doi: 10.3389/fpls.2023.1131315 (PMC10203591; doi:10.3389/fpls.2023.1131315)
Supplement: Supplementary file 1 [file DataSheet_1.docx]

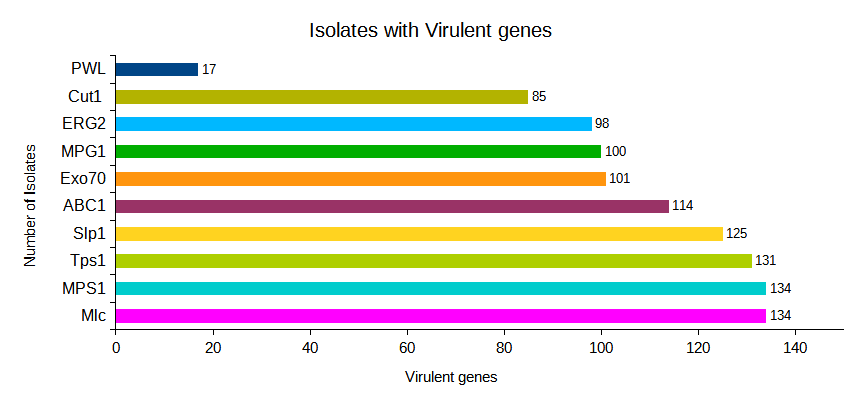


**Suppl. Fig. 1. Distribution of virulent genes in *Magnaporthe* isolates infecting millets**


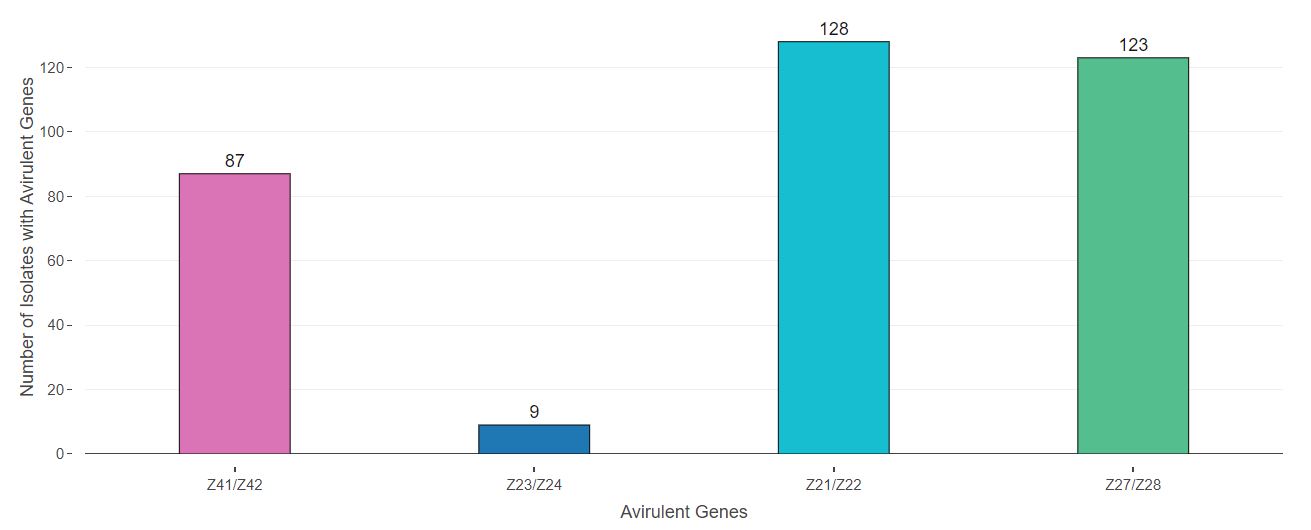


**Suppl. Fig. 2. Distribution of avirulent genes in *Magnaporthe* isolates infecting millets**

**
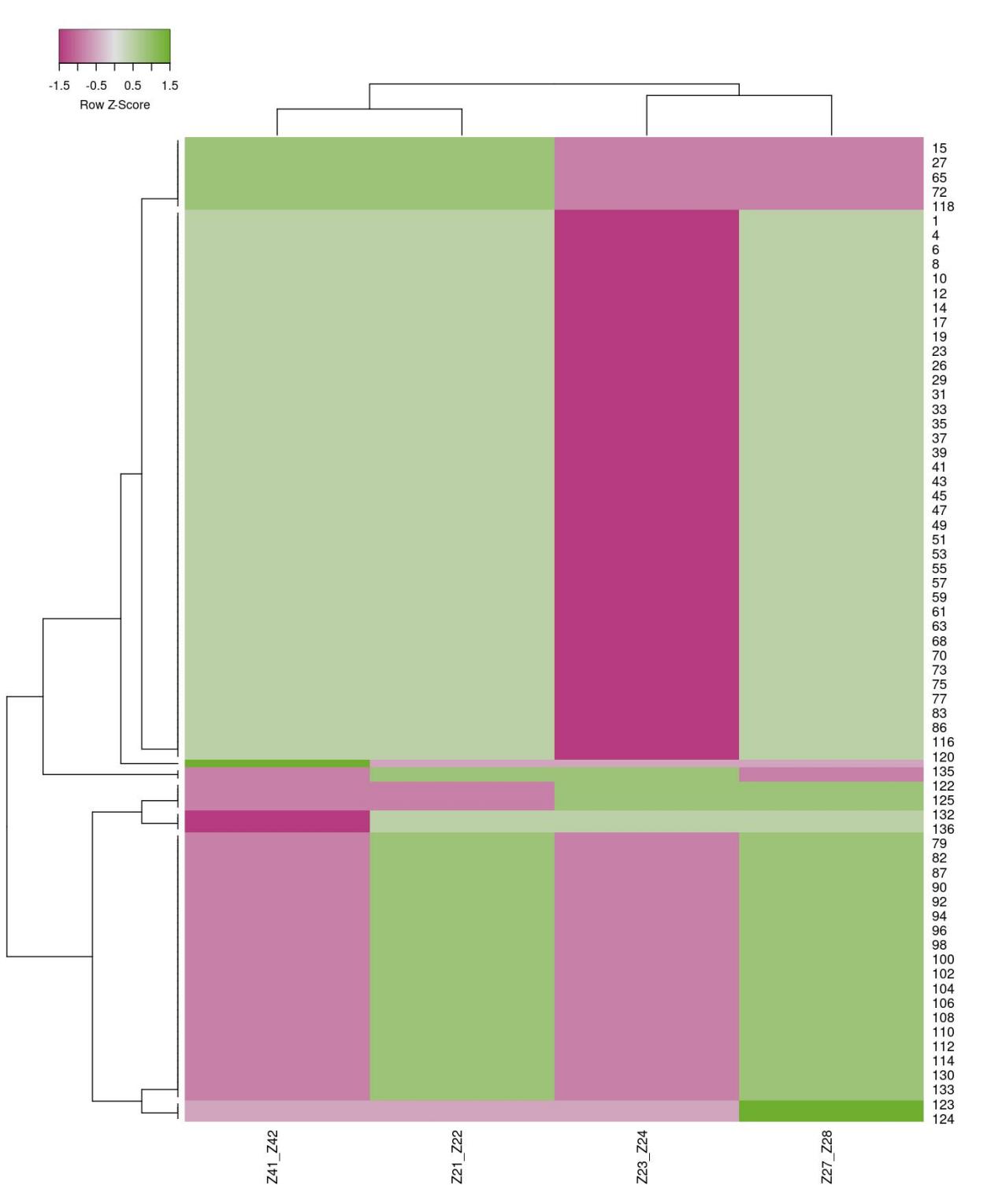
**

**Suppl. Fig. 3. Heat map for avirulence genes of *Magnaporthe* infecting millets**


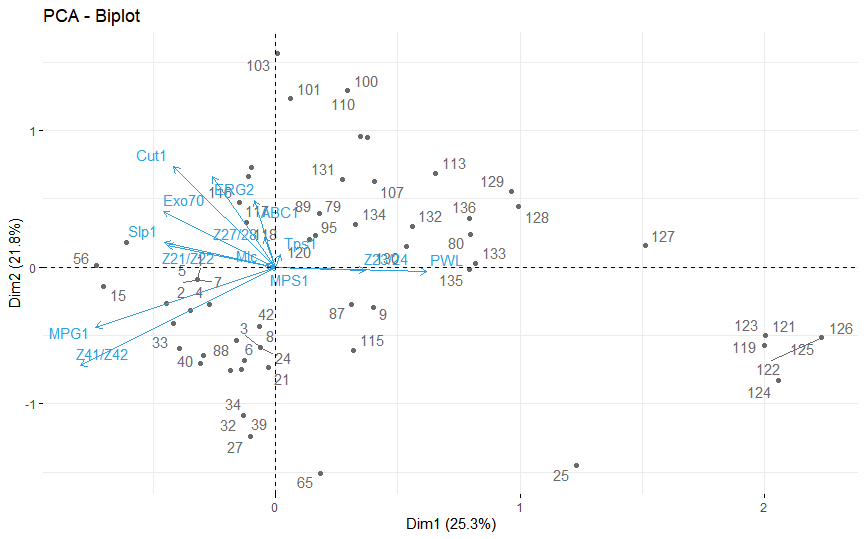


**Suppl. Fig. 4. Isolate wise distribution of virulence genes of *Magnaporthe* infecting millets**


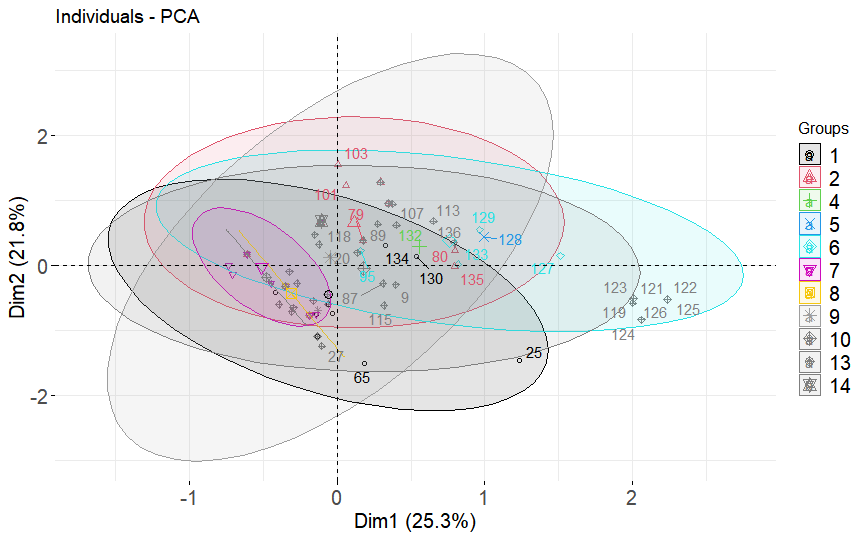


**Suppl. Fig. 5. Geographical distribution of virulence genes of *Magnaporthe* infecting millets**
