# Supplementary material for: Exploring the diversity of virulence genes in the Magnaporthe population infecting millets and rice in India
Source: Front Plant Sci. 2023 May 9;14:1131315. doi: 10.3389/fpls.2023.1131315 (PMC10203591; doi:10.3389/fpls.2023.1131315)
Supplement: Supplementary file 2 [file Table_1.docx]

**Supplementary Table 1. Details of gene bank accession number of** *Magnaporthe* **isolates submitted to NCBI**

| **SL.NO** | **ISOLATE** | **CROP** | **ITS accession number** |
| --- | --- | --- | --- |
| 1 | FMPg1 | Finger millet | MW423031 |
| 2 | FMPg2 | Finger millet | MW423032 |
| 3 | FMPg3 | Finger millet | MW423033 |
| 4 | FMPg4 | Finger millet | MW423034 |
| 5 | FMPg5 | Finger millet | MW423035 |
| 6 | FMPg6 | Finger millet | MW423036 |
| 7 | FMPg7 | Finger millet | MW423037 |
| 8 | FMPg8 | Finger millet | MW423038 |
| 9 | FMPg9 | Finger millet | MW423039 |
| 10 | FoxMPs1 | Foxtail millet | MW423040 |
| 11 | FoxMPs2 | Foxtail millet | MW423041 |
| 12 | FoxMPs3 | Foxtail millet | MW423042 |
| 13 | FoxMPs4 | Foxtail millet | MW423043 |
| 14 | FoxMPs5 | Foxtail millet | MW423044 |
| 15 | FMPg10 | Finger millet | MW423045 |
| 16 | FMPg11 | Finger millet | MW423046 |
| 17 | FMPg12 | Finger millet | MW423047 |
| 18 | FMPg13 | Finger millet | MW423048 |
| 19 | FMPg14 | Finger millet | MW423049 |
| 20 | FMPg15 | Finger millet | MW423050 |
| 21 | FMPg16 | Finger millet | MW423051 |
| 22 | FMPg17 | Finger millet | MW423052 |
| 23 | FMPg18 | Finger millet | MW423053 |
| 24 | FMPg19 | Finger millet | MW423054 |
| 25 | FMPg20 | Finger millet | MW423055 |
| 26 | FMPg22 | Finger millet | MW423056 |
| 27 | FMPg23 | Finger millet | MW423057 |
| 28 | FMPg24 | Finger millet | MW423058 |
| 29 | FMPg25 | Finger millet | MW423059 |
| 30 | BMPg1 | Barnyard millet | MW423060 |
| 31 | BMPg2 | Barnyard millet | MW423061 |
| 32 | FMPg27 | Finger millet | MW423062 |
| 33 | FMPg28 | Finger millet | MW423063 |
| 34 | BMPg3 | Barnyard millet | MW423064 |
| 35 | FMPg29 | Finger millet | MW423065 |
| 36 | FoxMPs6 | Foxtail millet | MW423066 |
| 37 | FoxMPs7 | Foxtail millet | MW423067 |
| 38 | FMPg29 | Foxtail millet | MW423068 |
| 39 | FoxMPs8 | Foxtail millet | MW423070 |
| 40 | FoxMPs9 | Foxtail millet | MW423071 |
| 41 | FoxMPs10 | Foxtail millet | MW423072 |
| 42 | FoxMPs11 | Foxtail millet | MW423073 |
| 43 | FoxMPs12 | Foxtail millet | MW423074 |
| 44 | FMpg31 | *Eleusine coracana* | OM721829 |
| 45 | FMpg32 | *Eleusine coracana* | OM721830 |
| 46 | FMpg33 | *Eleusine coracana* | OM721831 |
| 47 | FMpg34 | *Eleusine coracana* | OM721832 |
| 48 | FMpg35 | *Eleusine coracana* | OM721833 |

**Supplementary Table 1. Details of gene bank accession number of** *Magnaporthe* **isolates submitted to NCBI (Contd…..)**

| **Sl. No** | **Isolate name** | **Crop** | **ITS accession number** |
| --- | --- | --- | --- |
| 49 | FMpg36 | *Eleusine coracana* | OM721834 |
| 50 | FMpg37 | *Eleusine coracana* | OM721835 |
| 51 | FMpg38 | *Eleusine coracana* | OM721836 |
| 52 | BMpg4 | *Echinochloa esculenta* | OM721837 |
| 53 | HBPP1 | *Pennisetum purpureum* | OM721838 |
| 54 | FMpg39 | *Eleusine coracana* | OM721839 |
| 55 | FMpg40 | *Eleusine coracana* | OM721840 |
| 56 | FMpg41 | *Eleusine coracana* | OM721841 |
| 57 | FMpg42 | *Eleusine coracana* | OM721842 |
| 58 | FoXMpg13 | *Setaria italica* | OM721843 |
| 59 | FoXMpg14 | *Setaria italica* | OM721844 |
| 60 | FoXMpg15 | *Setaria italica* | OM721845 |
| 61 | FoXMpg16 | *Setaria italica* | OM721846 |
| 62 | FoXMpg17 | *Setaria italica* | OM721847 |
| 63 | FoXMpg18 | *Setaria italica* | OM721848 |
| 64 | FoXMpg19 | *Setaria italica* | OM721849 |
| 65 | FoXMpg20 | *Setaria italica* | OM721850 |
| 66 | FoXMpg21 | *Setaria italica* | OM721851 |
| 67 | FoXMpg22 | *Setaria italica* | OM721852 |
| 68 | FMpg43 | *Eleusine coracana* | OM721853 |
| 69 | FMpg44 | *Eleusine coracana* | OM721854 |
| 70 | FMpg45 | *Eleusine coracana* | OM721855 |
| 71 | FMpg46 | *Eleusine coracana* | OM721856 |
| 72 | FoXMpg23 | *Setaria italica* | OM721857 |
| 73 | FoXMpg24 | *Setaria italica* | OM721858 |
| 74 | FoXMpg25 | *Setaria italica* | OM721859 |
| 75 | FMpg47 | *Eleusine coracana* | OM721860 |
| 76 | FMpg48 | *Eleusine coracana* | OM721861 |
| 77 | FMpg49 | *Eleusine coracana* | OM721862 |
| 78 | FMpg50 | *Eleusine coracana* | OM721863 |
| 79 | FMpg51 | *Eleusine coracana* | OM721864 |
| 80 | FMpg52 | *Eleusine coracana* | OM721865 |
| 81 | FMpg53 | *Eleusine coracana* | OM721866 |
| 82 | FMpg54 | *Eleusine coracana* | OM721867 |
| 83 | FMpg55 | *Eleusine coracana* | OM721868 |
| 84 | FMpg56 | *Eleusine coracana* | OM721869 |
| 85 | FMpg57 | *Eleusine coracana* | OM721870 |
| 86 | FMpg58 | *Eleusine coracana* | OM721871 |
| 87 | FMpg59 | *Eleusine coracana* | OM721872 |

**Supplementary Table 1. Details of gene bank accession number of *Magnaporthe* isolates submitted to NCBI (Contd…)**

| **SL. No** | **Isolate name** | **Crop** | **ITS accession number** |
| --- | --- | --- | --- |
| 88 | FMpg60 | *Eleusine coracana* | OM721873 |
| 89 | FMpg61 | *Eleusine coracana* | OM721874 |
| 90 | FoXMpg26 | *Setaria italica* | OM721875 |
| 91 | FoXMpg27 | *Setaria italica* | OM721876 |
| 92 | FoXMpg28 | *Setaria italica* | OM721877 |
| 93 | FoXMpg29 | *Setaria italica* | OM721878 |
| 94 | BPg2 | *Pennisetum glaucum* | OM721879 |
| 95 | BPg3 | *Pennisetum glaucum* | OM721880 |
| 96 | BPg4 | *Pennisetum glaucum* | OM721881 |
| 97 | BPg5 | *Pennisetum glaucum* | OM721882 |
| 98 | BPg6 | *Pennisetum glaucum* | OM721883 |
| 99 | BPg7 | *Pennisetum glaucum* | OM721884 |
| 100 | FMpg62 | *Eleusine coracana* | OM721885 |
| 101 | FMpg63 | *Eleusine coracana* | OM721886 |
| 102 | FMpg64 | *Eleusine coracana* | OM721887 |
| 103 | FMpg65 | *Eleusine coracana* | OM721888 |
| 104 | FoXMpg30 | *Setaria italica* | OM721889 |
| 105 | FoXMpg31 | *Setaria italica* | OM721890 |
| 106 | FoXMpg32 | *Setaria italica* | OM721891 |
| 107 | FoXMpg33 | *Setaria italica* | OM721892 |
| 108 | FoXMpg34 | *Setaria italica* | OM721893 |
| 109 | FoXMpg35 | *Setaria italica* | OM721894 |
| 110 | FoXMpg36 | *Setaria italica* | OM721895 |
| 111 | FoXMpg37 | *Setaria italica* | OM721896 |
| 112 | FMpg66 | *Eleusine coracana* | OM721897 |
| 113 | FMpg67 | *Eleusine coracana* | OM721898 |
| 114 | FMpg68 | *Eleusine coracana* | OM721899 |
| 115 | FMpg69 | *Eleusine coracana* | OM721900 |
| 116 | FMpg70 | *Eleusine coracana* | OM721901 |
| 117 | FMpg71 | *Eleusine coracana* | OM721902 |
| 118 | FMpg72 | *Eleusine coracana* | OM721903 |
| 119 | FMpg73 | *Eleusine coracana* | OM721904 |
| 120 | FMpg74 | *Eleusine coracana* | OM721905 |
| 121 | FMpg75 | *Eleusine coracana* | OM721906 |
| 122 | FMpg76 | *Eleusine coracana* | OM721907 |
| 123 | FMpg77 | *Eleusine coracana* | OM721908 |
| 124 | FMpg78 | *Eleusine coracana* | OM721909 |

**Supplementary Table 1. Details of gene bank accession number of *Magnaporthe* isolates submitted to NCBI (Contd….)**

| **SL. No** | **Isolate name** | **Crop** | **ITS accession number** |
| --- | --- | --- | --- |
| 125 | FMpg79 | *Eleusine coracana* | OM721910 |
| 126 | FMpg80 | *Eleusine coracana* | OM721911 |
| 127 | RPo1 | *Oryza sativa* | OM721931 |
| 128 | RPo2 | *Oryza sativa* | OM721932 |
| 129 | RPo3 | *Oryza sativa* | OM721933 |
| 130 | RPo4 | *Oryza sativa* | OM721934 |
| 131 | RPo5 | *Oryza sativa* | OM721935 |
| 132 | RPo6 | *Oryza sativa* | OM721936 |
| 133 | RPo7 | *Oryza sativa* | OM721937 |
| 134 | RPo8 | *Oryza sativa* | OM721938 |
| 135 | RPo9 | *Oryza sativa* | OM721939 |
| 136 | RPo10 | *Oryza sativa* | OM721940 |
